# The effect of smoking and other tobacco product use on perceptions of skin quality and health, approaches to skin care, and minimally invasive cosmetic procedures: A cross-sectional study

**DOI:** 10.18332/tid/207157

**Published:** 2025-08-22

**Authors:** Fatma Etgü, Emine Serap Yılmaz

**Affiliations:** 1 Department of Dermatology, Faculty of Medicine, Ordu University, Ordu, Türkiye; 2 Department of Pulmonology, Faculty of Medicine, Ordu University, Ordu, Türkiye

**Keywords:** smoking, electronic cigarette, hookah, skin quality, skin aging

## Abstract

**INTRODUCTION:**

It is well-established that smoking adversely affects the skin. This study aimed to compare skin properties, skin care product usage patterns, and the status of minimally invasive cosmetic procedures and self-evaluated skin quality in active smokers, ex-smokers, and non-smokers.

**METHODS:**

This cross-sectional study was conducted in Department of Dermatology, Faculty of Medicine, Ordu University, Türkiye in 2024, with participants aged 18–65 years. Data were collected through a validated self-administered questionnaire. Categorical data were analyzed using chi-squared or Fisher’s exact tests. Intergroup differences were examined using one-way analysis of variance with *post hoc* Bonferroni tests. Correlations between smoking duration, daily cigarette consumption, and total skin quality scores were assessed using Pearson and Spearman correlation analyses and modeled with linear regression. Receiver Operating Characteristic (ROC) analysis was used to assess the skin quality score’s ability to distinguish smokers from non-smokers. Logistic regression analysis was conducted to examine the association between smoking and skin quality. Structural Equation Modeling (SEM) was used to explore the relationships between smoking, age, gender, and skin quality.

**RESULTS:**

The study included 286 men and 393 women. Active smokers had worse skin quality, with more wrinkles, spots, and pigmentation (p<0.001). E-cigarette users showed poorer skin in the forehead, around the eyes, mustache, mouth, neck, and back (p=0.007–0.034). Hookah use was linked to worse skin and more spots on the back (p=0.004 and 0.009). Average skin quality scores were 25.47 for active smokers, 27.35 for ex-smokers, and 32.1 for non-smokers. Skin quality declined as smoking duration and daily cigarette count increased (p=0.00). Active smokers more frequently received neurotoxin injections and mesotherapy for skin spots (p=0.006 and 0.026).

**CONCLUSIONS:**

This study confirms the detrimental effects of smoking – including e-cigarette and hookah use – on skin. These findings may serve as motivation for smoking cessation efforts.

## INTRODUCTION

As the demographic shifts toward an older population and life expectancy continues to rise, the process of aging becomes unavoidable, leading to a heightened focus on its implications for all humans. The skin, being the body’s largest organ and constantly exposed to external harmful factors, exhibits the most apparent signs of aging. Skin aging is characterized as a gradual deterioration in the skin’s functional and regenerative abilities over time^1^.

The following findings can be observed in skin aging: reduced skin thickness; fewer sweat glands and dermal blood vessels; a reduction in the density and functionality of melanocytes and Langerhans cells; degeneration of the elastic fibers; decreased collagen synthesis; and variations in bone and fat density^2^. Two mechanisms contribute to skin aging. The first is intrinsic aging, which cannot be delayed or stopped and is determined by genetics, race, age, gender, and physiological processes. The second is extrinsic aging, influenced by various external factors, including sunlight, smoking, and dietary habits^3^. To delay aging, protection from these factors is crucial.

The effects of smoking on the skin have been previously investigated. A previous study found that smoking was associated with skin aging, and it increased perioral wrinkles in older women, but not in men^4^. Smoking increases the risk of delayed wound healing, allergic contact dermatitis, hidradenitis suppurativa, acne, androgenic alopecia, lupus erythematosus, polymorphous photodermatosis, and skin cancers (actinic keratosis, squamous cell carcinoma, basal cell carcinoma, melanoma, and anogenital cancer)^5^.

The demand for cosmetic procedures is growing due to the increasing variety of cosmetic surgeries, easier access, lower prices, and widespread use of social media. Interest is more common among women, although interest among men is also rising, and the age of initiation of cosmetic surgery is gradually decreasing^6,7^. The desire to look more attractive in photos is the biggest driver of interest in cosmetic surgery. Reasons include the increasing use of social media, fascination with people portrayed in the media, feelings of inadequacy, and the desire for beautiful photos that lead to more interactions^7^.

The aim of this study was to compare skin properties, skin care product usage patterns, and the status of minimally invasive cosmetic procedures and self-evaluated skin quality based on smoking habits.

## METHODS

### Study design and participants

This is a cross-sectional study conducted in the Department of Dermatology outpatient clinic of Ordu University, Türkiye, between November 2023 and May 2024. The study consisted of 679 healthy individuals. The participants completed a self-administered questionnaire. The patients were recruited from the general dermatology clinic of Ordu University, with insignificant diseases, such as warts and calluses, and their healthy relatives and provided written informed consent before enrollment.

Participants eligible for inclusion in this cross-sectional study were adults aged between 18–65 years. All participants were required to provide informed consent and possess the ability to read, comprehend, and independently complete the questionnaire.

Exclusion criteria encompassed a history of dermatological conditions such as eczema, psoriasis, acne vulgaris, or vitiligo, as well as any systemic diseases known to affect skin quality, including but not limited to diabetes mellitus, thyroid disorders, autoimmune diseases, and chronic kidney or liver disorders. Participants with incomplete or inconsistent responses on the questionnaire were also excluded from the study. Prior to the study, ethical approval was obtained from the Ethics Committee of Ordu University (Approval number: 2023/289; Date: 10 November 2023).

A simple random sampling method was used to determine the study population. The first 13 questions of the questionnaire assessed demographic data and the frequency and duration of tobacco and alcohol use. Questions 14–36 focused on skin type, the use of skin care products, and cosmetic procedure applications. Question 37 asked participants to evaluate their own 12 body parts.

**Figure 1:**
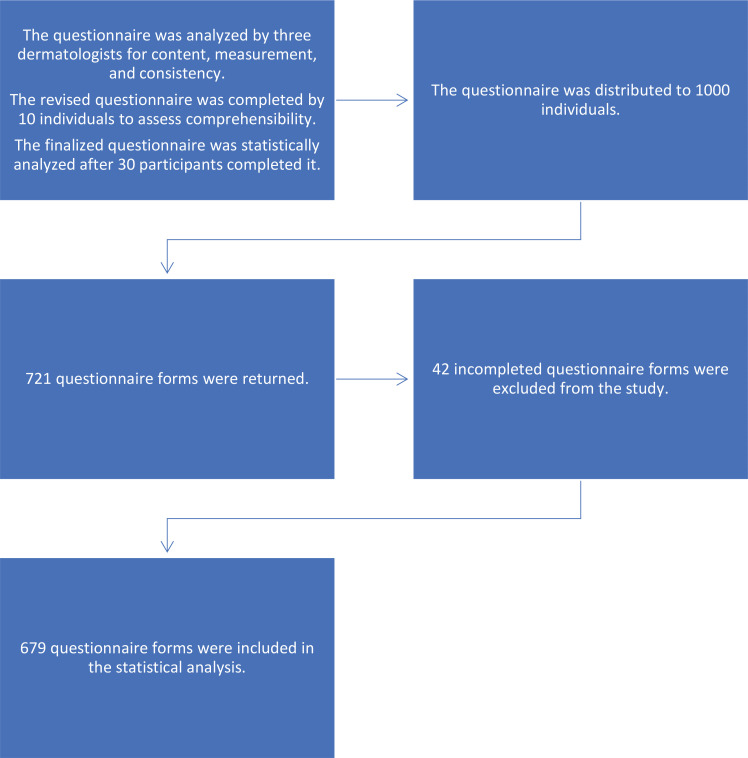
Flow chart of the study

While preparing the study questionnaire, questions were formulated by examining previous studies and existing academic knowledge and current literature and previous studies. The questionnaire was analyzed by three dermatologists for content, measurement, and consistency. The questionnaire was revised according to their suggestions. The revised questionnaire was distributed to 10 people to analyze its comprehensibility and finalized according to their comments and suggestions. The finalized questionnaire was statistically analyzed after 30 participants completed it (Figure 1). The questionnaire was validated with reliability analysis (Cronbach’s alpha). The analysis shows excellent internal consistency among the 12 skin quality measurement items, with a Cronbach’s alpha of 0.859. This indicates high reliability of the scale as a measurement tool. The scale demonstrates strong internal consistency and appears to reliably measure whatever skin quality construct it was designed to assess.

Demographic information (gender, age, education level, marital status, employment status, place of residence, and monthly income), smoking status, use of electronic cigarettes, status of waterpipe (shisha or hookah) smoking, alcohol usage, and skin type (dry, oily, normal, combination, or sensitive) were assessed. Additional questions about facial or body lesions (discoloration, freckles, redness/telangiectasia, acne, and scars), and frequency of makeup applications (always, frequently, on special occasions, and never). The participants were also asked about regular skin care product usage, use of dietary supplements for skin health, use of hand/face/body moisturizers, frequency of moisturizer use (every day, 1–2 times per week, as needed, or never), use of anti-aging products, regular skin care routines (masks, peels, or skin cleansing), and sun protection methods. The participants reported their sun protection practices, including protective clothing (hats, glasses, etc.), avoiding outdoor activities during peak hours (between 10:00 and 16:00), and sunscreen usage (only in summer, year-round, or never). The frequency of sunscreen reapplication (once daily, twice daily, or every 2–3 hours) and areas of application (face, hands, arms, neck, and décolleté) were also recorded. Other questions addressed cosmetic procedures such as neurotoxin injections, fillers, mesotherapy, and platelet-rich plasma injections, as well as the age of first application.

Smoking status and electronic cigarette use were categorized as active smokers, ex-smokers, and non-smokers. The duration of cigarette use and the quantity smoked were also assessed.

### Evaluation of skin quality

Twelve body parts (forehead, between the eyebrows, around the eyes, cheeks, mustache area, around the mouth, chin, neck, décolleté, back, shoulders, and back of the hands/forearms) were evaluated by the participants in terms of skin quality (good/bad), wrinkle status (present/absent), and skin discoloration (present/absent). Scoring was as follows: good skin quality = 1 point, bad skin quality = 0 points; no wrinkles = 1 point, wrinkles = 0 points; and no spots on the skin = 1 point, spots present = 0 points. A total skin score was calculated, ranging from 0 to 36, with higher scores indicating better perceived skin quality.

### Sample calculation

Using Open Epi Info (version 3.01), a sample size calculation was conducted based on the population of individuals aged 18–64 years (n=506378) within Ordu Province^8^. It was determined that a minimum of 305 participants would be required to achieve a 95% confidence interval. The smoking prevalence in Ordu, estimated at 27.3%, was factored into the calculation.

### Statistical analysis

Statistical analyses were conducted using IBM^®^ SPSS^®^ version 27 (Armonk, NY: IBM Corp., USA)^9^. The normality of variable distributions was assessed using the Kolmogorov-Smirnov and Shapiro-Wilk tests. Descriptive data are presented as mean ± standard deviation for continuous data and as frequencies and percentages for categorical variables. A receiver operating characteristic (ROC) analysis was conducted to evaluate the ability of the total skin quality score to distinguish between smokers and non-smokers. Categorical data were analyzed using either Pearson’s chi-squared or Fisher’s exact tests. Intergroup differences were examined using one-way analysis of variance with *post hoc* Bonferroni tests. The relationship between the duration of smoking duration and the total skin score related to the number of cigarettes was examined by Pearson/Spearman rho correlation analysis and modelled by linear regression analysis. The logistic regression analysis examined the association between smoking status and skin quality, including wrinkles and skin spots.

In the analysis of the data, structural equation modelling (SEM) was used to examine the causal relationships between variables. The model was estimated by maximum likelihood (ML) method and the effects of age groups, smoking status and gender on skin quality were evaluated. Model fit was tested with indices such as Comparative Fit Index (CFI), Tucker-Lewis Index (TLI), Root Mean Square Error of Approximation (RMSEA) and Standardized Root Mean Square Residual (SRMR)^10^. Statistical significance was set at p<0.05.

Power analysis was calculated using G*Power ver. 3.1.9.7 software^11^. In the calculation made with the sample numbers in the groups (Group 1: 288; Group 2: 331; Group 3: 80), the effect size value (effect size; f) was determined as 0.64 and the actual power was calculated as 100%^10^. According to Cohen, it is predicted that there should be at least 80% power in a scientific study and according to this criterion, the study will be completed with an appropriate power (according to one-way ANOVA and *post hoc* test selection, the critical f value was found to be 0.190 and the effect size was taken as 0.64).

## RESULTS

The validated questionnaire was distributed to 1000 individuals, of whom 721 returned the questionnaire; 42 completed questionnaires were excluded from the study because of missing or inappropriate results. The remaining 679 results were included in the statistical evaluation. The flowchart of the study is shown in Figure 1. The study population consisted of 286 males and 393 females. The age distribution of the study group was as follows: <20 (2.8%), 20–40 (63.2%), 41–60 (32.1%), and >60 years (1.9%); 4.6% were educated at primary school, and the rest were educated from at least high school; 60.4% were employed, and 80% were living in urban areas; and 32.9% of respondents had monthly earnings below the minimum wage. Smoking status was active, never, and ex-smoker in 42.4%, 45.8%, and 11.8%, respectively. E-cigarettes were actively used by 5.6%, never used by 87.2%, and 7.2% were ex-smokers; 9.3% were active hookah users, and 11.5% were ex-hookah users. The demographic characteristics of the participants are shown in Table 1.

**Table 1:** Sociodemographic characteristics of participants, a cross-sectional study, Ordu University, Türkiye, 2024 (N=679)

*Characteristics*	*n*	*%*
**Gender**		
Male	286	42.1
Female	393	57.9
**Age** (years)		
<20	19	2.8
20–40	429	63.2
41–60	218	32.1
>60	13	1.9
**Education level**		
Primary school	31	4.6
High school	116	17.1
University	521	78.3
**Employment status**		
Employed	410	60.4
Unemployed	269	39.6
**Residence**		
Urban	543	80.0
Rural	136	20.0
**Monthly income** (TL)		
>15000	237	34.9
15000–10000	176	25.9
<10000	266	39.2
**Smoking status**		
Active smoker	288	42.4
Never smoker	311	45.8
Ex-smoker	80	11.8
**Electronic cigarette use**		
Active smoker	38	5.6
Never smoker	592	87.2
Ex-smoker	49	11.8
**Hookah use**		
Active smoker	63	9.3
Never smoker	538	79.2
Ex-smoker	78	11.5
**Alcohol use**		
Active user	185	27.2
Never user	410	60.4
Former user	84	12.4
Duration of smoking (years), mean ± SD	5.95 ± 8.56	
Cigarettes smoked per day, mean ± SD	6.57 ± 8.40	

TL: 1000 Turkish Liras about US$25.

The comparison of skin type, skin symptoms, and skin care product use between tobacco product users and non-users is presented in Table 2. The results of the chi-squared analysis showed that active smokers and individuals who had quit smoking were more likely to have a dry skin type, whereas individuals who had never smoked were more likely to have a normal skin type (p=0.012). According to *post hoc* Bonferroni analyses, the prevalence of dry skin was significantly lower in non-smokers compared to active smokers (p=0.042) and former smokers (p=0.018). The prevalence of normal skin was significantly higher in non-smokers than in active smokers and former smokers (p=0.008 and p=0.003, respectively). The rate of regular skin care was significantly lower in former smokers compared to never smokers and active smokers (p=0.025 and p=0.004, respectively). The frequency of body moisturizer use was significantly higher in non-smokers than in active smokers and former smokers (p=0.038 and p=0.012, respectively). Regarding the frequency of moisturizer use, daily use was more common among non-smokers, whereas never use was more prevalent among active smokers (p=0.022 and p=0.005, respectively).

**Table 2:** Comparison of categorical variables among active smokers, non-smokers, and ex-smokers, a crosssectional study, Ordu University, Türkiye, 2024 (N=679)

*Variables*	*Active smokers (N=288)*	*Non-smokers (N=311)*	*Ex-smokers (N=80)*	p*
	*n (%)*			
**Skin type**				
Dry	62 (21.5)*	47 (15.1)	21 (26.3)*	**0.012**
Oily	55 (19.1)	55 (17.7)	21 (26.3)	
Mixed	101 (35.1)	99 (31.8)	23 (28.8)	
Sensitive	13 (4.5)	23 (7.4)	5 (6.3)	
Normal	57 (19.8)	87 (28.0)*	10 (12.5)	
**Regular skin care**				
Yes	93 (32.3)*	113 (36.3)*	15 (18.8)	**0.011**
No	195 (67.7)	198 (63.7)	65 (81.3)	
**Makeup application frequency**				
Everyday	65 (22.6)	67 (21.5)	11 (13.8)	0.260
Mostly	34 (11.8)	41 (13.2)	7 (8.8)	
Special occasions	42 (14.6)	60 (19.3)	17 (21.3)	
Never	147 (51.0)	143 (46.0)	45 (56.3)	
**Dietary supplement use**				
Yes	32 (11.1)	36 (11.6)	3 (3.8)	0.111
No	256 (88.9)	275 (88.4)	77 (96.3)	
**Hand moisturizer use**				
Yes	161 (55.9)	190 (61.1)	48 (60.0)	0.423
No	127 (44.1)	121 (38.9)	32 (40.0)	
**Facial moisturizer use**				
Yes	146 (50.7)	165 (53.1)	35 (43.8)	0.330
No	142 (49.3)	146 (46.9)	45 (56.3)	
**Body moisturizer use**				
Yes	83 (28.8)	112 (36.0)*	17 (21.3)	**0.020**
No	205 (71.2)	199 (64.0)	63 (78.8)	
**Frequency of moisturizer use**				
Every day	91 (31.6)	112 (36.0)*	20 (25.0)	**0.014**
1–2 times per week	44 (15.3)	46 (14.8)	9 (11.3)	
As needed	63 (21.9)	91 (29.3)	27 (33.8)*	
Never	90 (31.3)*	62 (19.9)	24 (30.0)*	
**Anti-aging product use**				
Yes	46 (16.0)	35 (11.3)	6 (7.5)	0.072
No	242 (84.0)	276 (88.7)	74 (92.5)	
**Sun protection status**				
Yes	198 (68.8)	226 (72.7)	58 (72.5)	0.544
No	90 (31.3)	85 (27.3)	22 (27.5)	
**Frequency of sunscreen use**				
Only summer	106 (36.8)	106 (34.1)	37 (46.3)	0.062
Year-round	92 (31.9)	118 (37.9)	17 (21.3)	
Never	90 (31.3)	87 (28.0)	26 (32.5)	
**Frequency of sunscreen use**				
Never	88 (30.6)	85 (27.3)	27 (33.8)	0.192
1 time per day	147 (51.0)	171 (55.0)	47 (58.8)	
2 times per day	33 (11.5)	41 (13.2)	4 (5.0)	
Every 1–2 hours	20 (6.9)	14 (4.5)	2 (2.5)	

* Pearson’s chi-squared test, statistically significant at p<0.05.

The frequency of minimally invasive cosmetic procedures is shown in Table 3. According to the chi-squared analysis results, among the participants who had botulinum toxin injections, 59.5% were active smokers (p=0.006). Similarly, the majority of those who had undergone mesotherapy for skin pigmentations were active smokers (p=0.026). In *post hoc* Bonferroni analyses, the frequency of neurotoxin administration was significantly higher in active smokers compared to non-smokers (p=0.003) and former smokers (p=0.032). Additionally, the rate of hyperpigmentation mesotherapy was significantly higher in active smokers than in non-smokers (p=0.018).

**Table 3:** Comparison of smoking status according to minimally invasive procedure rates, a cross-sectional study, Ordu University, Türkiye, 2024 (N=679)

*Variables*	*Active smokers (N=288)*	*Non-smokers (N=311)*	*Ex-smokers (N=80)*	p*
	*n (%)*			
**Neurotoxin injection**				
Yes	44 (15.3)*	25 (8.0)	5 (6.3)	**0.006**
No	244 (84.7)	286 (92.0)	75 (93.8)	
**Filler**				
Yes	13 (4.5)	14 (4.5)	2 (2.5)	0.706
No	275 (95.5)	297 (95.5)	78 (97.5)	
**Hyperpigmentation mesotherapy**				
Yes	9 (3.1)*	2 (0.6)	0 (0)	**0.026**
No	279 (96.9)	309 (99.4)	80 (100)	
**Antiaging mesotherapy**				
Yes	7 (2.4)	7 (2.3)	1 (1.3)	0.815
No	281 (97.6)	304 (97.7)	79 (98.8)	
**Plasma rich platelet**				
Yes	10 (3.5)	6 (1.9)	1 (1.3)	0.360
No	278 (96.5)	305 (98.1)	79 (98.8)	

* Pearson’s chi-squared test, statistically significant at p<0.05.

Tables 4 and 5 present a comparison of perceived skin quality, wrinkles, and blemishes on 12 body regions between active smokers, ex-smokers, and never smokers. The chi-squared analysis results showed that compared to individuals who had never smoked, active smokers exhibited poorer skin quality, with more wrinkles, spots, and pigmentation on all 12 body areas (p<0.001). According to *post hoc* Bonferroni analyses, skin parameter values were significantly worse in active smokers compared to non-smokers across all body regions (p<0.001). Former smokers exhibited intermediate values, falling between those of non-smokers and active smokers (p<0.001).

**Table 4:** Comparison of perceived skin quality, wrinkles, and blemishes across 12 body areas between active smokers, non-smokers, and ex-smokers, a cross-sectional study, Ordu University, Türkiye, 2024 (N=679)

*Variables*	*Active smokers (N=288)*	*Non-smokers (N=311)*	*Ex-smokers (N=80)*	*p*
	*n (%)*			
**Forehead skin quality**				
Good	105 (36.5)	239 (76.8)	38 (47.5)	**<0.001**
Bad	183 (63.5)*	72 (23.2)	42 (52.5)*	
**Forehead wrinkles**				
Present	178 (61.8)*	107 (34.4)	41 (51.3)*	**<0.001**
Absent	110 (38.2)	204 (65.6)	39 (48.8)	
**Forehead blemishes**				
Present	69 (24.0)*	41 (13.2)	24 (30.0)*	**<0.001**
Absent	219 (76.0)	270 (86.8)	56 (70.0)	
**Glabella skin quality**				
Good	140 (48.6)	273 (87.8)	44 (55.0)	**<0.001**
Bad	148 (51.4)*	38 (12.2)	36 (45.0)*	
**Glabella wrinkles**				
Present	138 (47.9)*	67 (21.5)	35 (43.8)*	**<0.001**
Absent	150 (52.1)	244 (78.5)	45 (56.3)	
**Glabella blemishes**				
Present	36 (12.5)*	8 (2.6)	9 (11.3)*	**<0.001**
Absent	252 (87.5)	303 (97.4)	71 (88.8)	
**Eye area skin quality**				
Good	98 (34.0)	246 (79.1)	36 (45.0)	**<0.001**
Bad	190 (66.0)*	65 (20.9)	44 (55.0)*	
**Wrinkles around the eyes**				
Present	183 (63.5)*	109 (35.0)	45 (56.3)*	**<0.001**
Absent	105 (36.5)	202 (65.0)	35 (43.8)	
**Blemishes around the eye**				
Present	62 (21.5)*	31 (10.0)	16 (20.0)*	**<0.001**
Absent	226 (78.5)	280 (90.0)	64 (80.0)	
**Cheek skin quality**				
Good	137 (47.6)	250 (80.4)	44 (55.0)	**<0.001**
Bad	151 (52.4)*	61 (19.6)	36 (45.0)*	
**Cheek wrinkles**				
Present	38 (13.2)*	13 (4.2)	10 (12.5)*	**<0.001**
Absent	250 (86.8)	298 (95.8)	70 (87.5)	
**Cheek blemishes**				
Present	146 (50.7)*	91 (29.3)	34 (42.5)*	**<0.001**
Absent	142 (49.3)	220 (70.7)	46 (57.5)	
**Moustache area skin quality**				
Good	185 (64.2)	284 (91.3)	56 (70.0)	**<0.001**
Bad	103 (35.8)*	27 (8.7)	24 (30.0)*	
**Moustache area wrinkles**				
Present	61 (21.2)*	24 (7.7)	13 (16.3)*	**<0.001**
Absent	227 (78.8)	287 (92.3)	67 (83.8)	
**Moustache area blemishes**				
Present	56 (19.4)*	31 (10.0)	19 (23.8)*	**<0.001**
Absent	232 (80.6)	280 (90.0)	61 (76.3)	
**Skin quality around the mouth**				
Good	174 (60.4)	281 (90.4)	51 (63.8)	**<0.001**
Bad	114 (39.6)*	30 (9.6)	29 (36.3)*	
**Wrinkles around the mouth**				
Present	75 (26.0)*	27 (8.7)	16 (20.0)*	**<0.001**
Absent	213 (74.0)	284 (91.3)	64 (80.0)	
**Blemishes around the mouth**				
Present	54 (18.8)*	23 (7.4)	13 (16.3)*	**<0.001**
Absent	234 (81.3)	288 (92.6)	67 (83.8)	
**Chin skin quality**				
Good	211 (73.3)	270 (86.8)	65 (81.3)	**<0.001**
Bad	77 (26.7)*	41 (13.2)	15 (18.8)*	
**Chin wrinkles**				
Present	29 (10.1)*	11 (3.5)	7 (8.8)	**0.006**
Absent	259 (89.9)	300 (96.5)	73 (91.3)	
**Chin blemishes**				
Present	57 (19.8)*	34 (10.9)	10 (12.5)	**0.008**
Absent	231 (80.2)	277 (89.1)	70 (87.5)	

Pearson’s chi-squared test used and p<0.05 considered significant.

* Indicates statistical significance.

**Table 5:** Comparison of perceived skin quality, wrinkles, and blemishes across 12 body parts between active smokers, non-smokers, and ex-smokers, a cross-sectional study, Ordu University, Türkiye, 2024 (N=679)

*Variables*	*Active smokers (N=288)*	*Non-smokers (N=311)*	*Ex-smokers (N=80)*	p*
	*n (%)*			
**Neck skin quality**				
Good	196 (68.1)	294 (94.5)	69 (86.3)	**<0.001**
Bad	92 (31.9)*	17 (5.5)	11 (13.8)*	
**Neck wrinkles**				
Present	73 (25.3)*	28 (9.0)	12 (15.0)*	**<0.001**
Absent	215 (74.7)	283 (91.0)	68 (85.0)	
**Neck blemishes**				
Present	36 (12.5)*	13 (4.2)	5 (6.3)	**0.001**
Absent	252 (87.5)	298 (95.8)	75 (93.8)	
**Décolleté skin quality**				
Good	226 (78.5)	292 (93.9)	64 (80.0)	**<0.001**
Bad	62 (21.5)*	19 (6.1)	16 (20.0)*	
**Décolleté wrinkles**				
Present	34 (11.8)*	12 (3.9)	10 (12.5)*	**0.001**
Absent	254 (88.2)	299 (96.1)	70 (87.5)	
**Décolleté blemishes**				
Present	40 (13.9)*	11 (3.5)	9 (11.3)*	**<0.001**
Absent	248 (86.1)	300 (96.5)	71 (88.7)	
**Back skin quality**				
Good	218 (75.7)	286 (92.0)	60 (75.0)	**<0.001**
Bad	70 (14.3)*	25 (8.0)	20 (25.0)*	
**Back wrinkles**				
Present	15 (5.2)*	4 (1.3)	1 (1.3)	**0.011**
Absent	273 (94.8)	307 (98.7)	79 (98.7)	
**Back blemishes**				
Present	69 (24.0)*	33 (10.6)	17 (21.3)*	**<0.001**
Absent	219 (76.0)	278 (89.4)	63 (78.7)	
**Shoulder skin quality**				
Good	188 (65.3)	295 (94.9)	61 (76.3)	**<0.001**
Bad	100 (34.7)*	16 (5.1)	19 (23.8)*	
**Shoulder wrinkles**				
Present	14 (4.9)*	3 (1.0)	1 (1.3)	**0.009**
Absent	274 (95.1)	308 (99.0)	79 (98.7)	
**Shoulder blemishes**				
Present	95 (33.0)*	20 (6.4)	16 (20.0)*	**<0.001**
Absent	193 (67.0)	291 (93.6)	64 (80.0)	
**Skin quality on the back of the hand/forearm**				
Good	180 (62.5)	285 (91.6)	57 (71.3)	**<0.001**
Bad	108 (37.5)*	26 (8.4)	23 (28.7)*	
**Wrinkles on the back of the hand/forearm**				
Present	36 (12.5)*	15 (4.8)	6 (7.5)	**0.003**
Absent	252 (87.5)	296 (95.2)	74 (92.5)	
**Blemishes on the back of the hand/forearm**				
Present	89 (30.9)*	28 (9.0)	19 (23.8)*	**<0.001**
Absent	199 (69.1)	283 (91.0)	61 (76.3)	

* Pearson’s chi-squared test, statistically significant at p<0.05.

The chi-squared analyses of e-cigarette and hookah use were as follows. Active electronic cigarette (e-cigarette) users had worse skin quality in the glabellar area, around the eyes, the mustache area, around the mouth, the neck, and the back region (p=0.026, p=0.034, p=0.011, p=0.021, p=0.039, and p=0.007, respectively). They also exhibited more pigmentation in the glabellar area and on the back (p=0.041 and p=0.07, respectively). Hookah users were found to have poorer back skin quality and increased pigmentation on the back (p=0.004 and p=0.009, respectively).

**Figure 2:**
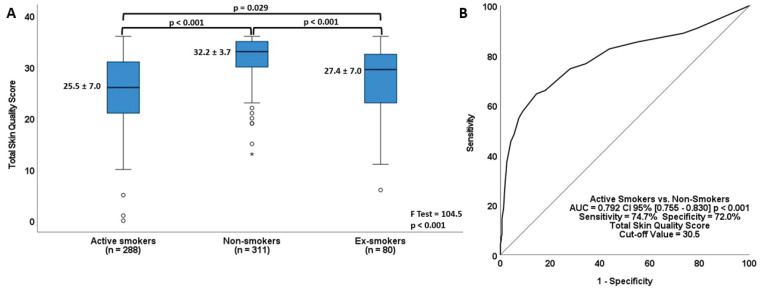
Relationship between smoking status and total skin quality scores: A) Comparison of total skin quality scores by smoking status (one-way ANOVA followed by Bonferroni post hoc test); B) Estimation of the cut-off value at which active smokers begin to experience a deterioration in total skin quality score (ROC analysis)

The total skin quality score was 25.47 ± 7.01, 27.35 ± 6.94, and 32.1 ± 3.71 for active smokers, ex-smokers, and never smokers, respectively. Total skin quality scores were also significantly worse in active smokers compared to never smokers (Figure 2A). A ROC analysis was performed to assess whether the total skin quality score could differentiate active smokers from never smokers, and to identify the threshold at which skin quality begins to decline (Figure 2B). The analysis yielded an area under the curve value of 0.792 (95% CI: 0.755–0.830). The cut-off value for the total skin quality score in active smokers was determined to be 30.5, with a sensitivity of 74.7% and a specificity of 72.0%.

The participants who used electronic cigarettes had worse total skin quality scores compared to those who had never used e-cigarettes (25.9 ± 6.28 vs 28.95 ± 6.54) (p=0.016). However, hookah use did not significantly affect total skin quality.

There was an inverse relationship between total skin quality and the number of cigarettes.

**Figure 3:**
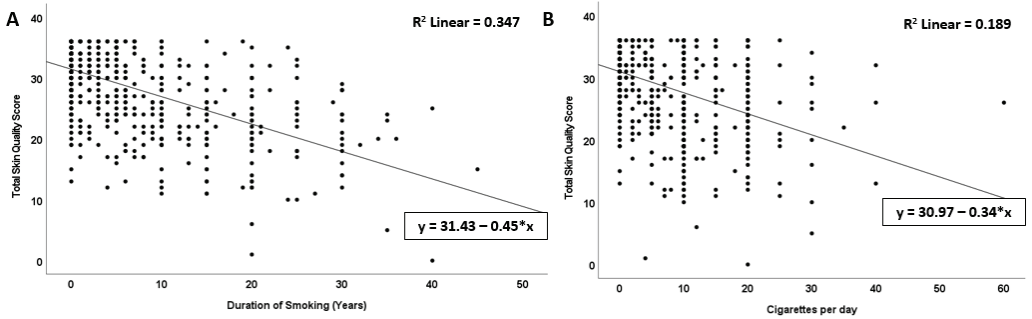
Correlation and regression analysis between smoking habits and total skin quality score: A) Analysis by smoking duration; B) Analysis by the number of cigarettes smoked per day

The regression model explained 34.7% of the total skin quality variance [R^2^=0.347, F(1, 676)=359.78, p<0.001]. This indicates that the duration of smoking has a moderate effect on skin quality. Each one-year increase in smoking duration is associated with a mean decrease in skin quality of 0.45 units. This effect is statistically significant (Beta= -0.589, p<0.001, 95% CI: -0.50 – -0.40), Constant term=31.43, p<0.001 (Figure 3A).

Linear regression analysis to examine the relationship between the number of cigarettes smoked per day and skin quality showed that cigarette consumption had a statistically significant negative effect on skin quality (B= -0.34, p<0.001). The model explained approximately 19% of the variance in skin quality (R^2^=0.189, Figure 3B).

The logistic regression analysis examined the association between smoking status and skin quality, including wrinkles and skin spots. The final model identified seven significant predictors, with Nagelkerke R^2^=0.31, indicating a moderate effect size. The model correctly classified 72.16% of cases, with higher accuracy for non-smokers (79.28%) than active smokers (62.5%).

Participants with good forehead skin quality were less likely to be smokers (B= -0.70, SE=0.22, p=0.001). The odds of being a smoker were 2.02 times lower (OR=2.02; 95% CI: 1.31–3.12) for those with good skin quality compared to those with poor quality. The presence of forehead skin spots was associated with a reduced likelihood of smoking (B= -0.64, SE=0.27, p=0.016, OR=1.90; 95% CI: 1.12–3.20). Participants with skin spots in the eyebrow area were 3.17 times more likely to be smokers (B=1.15, SE=0.36, p=0.001, OR=3.17; 95% CI: 1.56–6.47). Good skin quality around the eyes was linked to a lower likelihood of smoking (B= -0.84, SE=0.21, p<0.001, OR=2.33; 95% CI: 1.54–3.51). Better cheek skin quality was associated with a marginally reduced smoking likelihood (B= -0.41, SE=0.21, p=0.046, OR=1.51, 95% CI: 1.01–2.25). Good neck skin quality significantly predicted lower odds of smoking (B= -0.89, SE=0.26, p=0.001, OR=2.43; 95% CI: 1.45–4.09). The presence of skin spots (present) on the shoulders was strongly associated with higher odds of smoking (B=1.13, SE=0.24, p<0.001, OR=3.10; 95% CI: 1.94–4.93). The constant (intercept) was significant (B=1.35; SE=0.27, p<0.001), indicating baseline smoking odds when all predictors are zero.

Structural equation modelling (SEM) was used to examine the effect of smoking status (yes vs no), age groups (reference: <20, vs 20–40, 41–60 and >60 years) and gender (female vs male) on skin score. According to the results of the analysis, it was carried out to examine the effects of age groups, smoking status and gender on total skin quality. The model was estimated by maximum likelihood (ML) method and tested on a sample of 679 participants. The model fit indices showed an excellent fit (CFI=1.000, TLI=1.000, RMSEA=0.000, SRMR=0.000). The model explained 29.5% of the variance in skin quality (R^2^=0.295).

The findings regarding age groups showed that the age group of 41–60 years (compared to the <20 years reference group) led to a significant decrease in skin quality (β= -0.255, p=0.007). In the ≥60 years age group, this effect was even more pronounced (β= -0.192, p<0.001). Smoking had the strongest negative effect on skin quality (β= -0.449, p<0.001). In terms of gender, women reported lower skin quality compared to men (β= -0.136, p<0.001).

## DISCUSSION

Skin aging is characterized by dryness, decreased epidermal and dermal thickness, wrinkle formation, cutaneous lesions, irregular pigmentations, age spots, sagging, hair greying, and inappropriate wound healing^12-15^. As the most visible organ of the body, aging-related changes in the skin have a significant impact on individuals’ social lives and well-being^3^.

In a recent study, the authors investigated the skin aging characteristics of those perceived as elderly and found that skin tone and pigmentation were the most significant factors influencing perceived age in younger individuals, while wrinkles and sagging were more critical in older individuals. In addition, the cheeks, eyes, and forehead were strongly associated with perceived age^16^. Another study revealed that older age, urban living, tobacco smoke exposure, dry skin type, increased sun exposure, and working in toxic environments increased the odds of skin aging^17^. In our study, smoking, a primary cause of aging, was associated with worse perceived skin quality, more wrinkles, and blemishes. Active smokers frequently had a dry skin type. Furthermore, despite ultraviolet (UV) exposure being another important factor in aging, our study showed that effective sun protection practices were uncommon. Interestingly, even active smokers who consistently protected themselves from UV light exhibited poor skin quality, indicating that UV protection cannot mitigate the detrimental effects of smoking.

A meta-analysis examining factors influencing skin aging revealed conflicting findings regarding the impact of smoking on facial wrinkles and lentigines. However, all four studies included in the analysis found a dose-dependent correlation between smoking and wrinkles^18^. In our study, perceived skin quality decreased with increasing duration and quantity of smoking. It has been reported that smoking increases the activity of the matrix metalloproteinases-1 and -3, reducing collagen production, and it also increases the elastic fibers in the reticular dermis^4,5,19^. Chen et al.^20^ examined the effects of smoking on skin lipids, reporting that smoking disrupted lipid homeostasis, leaving the skin more vulnerable to aging and disorders. Our study corroborated these findings, as active smokers reported worse skin quality, more wrinkles, and increased skin pigmentation in all areas compared to never smokers.

The prevalence of e-cigarette use is rising, particularly among children and young adults. E-cigarette use can lead to nicotine dependence and associated adverse effects on the brain and lungs while also increasing susceptibility to tobacco and other substances^21,22^. In our study, the rate of active e-cigarette users was 5.6%, and quitters comprised 7.2% of participants. E-cigarette users had worse skin quality between the eyebrows and around the eyes and mouth, and on the neck and back. Total skin quality was significantly lower among active e-cigarette users than never users. Since e-cigarette sales are prohibited in our nation, our survey found a low rate of e-cigarette use. Furthermore, some of the skin changes brought on by e-cigarettes may be related to smoking because some of the patients who use them are either current smokers or have stopped. These factors suggest that a bigger sample size be used to examine the effects of e-cigarette use on skin health.

Waterpipes contain nicotine, carbon monoxide, polycyclic hydrocarbons, and other toxicants, potentially rendering them more harmful than cigarette smoke. A study of adolescents aged 12–16 years across 72 countries, found a hookah use prevalence of 6.8%, with the highest rates in European Union and Eastern Mediterranean countries^23^. In our study, the rate of active hookah users was 9.3%, with 11.5% classified as quitters. Hookah users exhibited poorer back skin quality and a higher rate of pigmentation in this area. However, it is important to note that some of the skin changes found in hookah users may be related to smoking, since participants who use hookah are likely to be active smokers and quit smoking, as well as passively exposed to cigarettes because they are in a smoking environment.

External factors influencing aging rarely act independently but often combine their effects^19^. A recent study investigating the combined effect of UV light and smoking on skin aging found that smoking and UVA1 exposure synergistically contributed to premature extrinsic aging^20^. In our study, 71% of the participants reported using some form of sun protection, with 46.4% using sunscreen, 33.4% using sunscreen year-round, and only 5.3% reapplying sunscreen every 2–3 hours. Our data underscore the inadequacy of current sun protection methods.

The demand for cosmetic procedures is increasing. A recent study using qualitative semi-structured interviews identified motivations for minimally invasive dermatologic cosmetic procedures and reported that, in addition to enhanced cosmetic appearance, patients sought to improve mental, emotional, and physical well-being, as well as social, work, and/or school performance^24^. According to the American Society for Dermatologic Surgery, the rate of cosmetic treatments doubled from 2013 to 2017, with wrinkle-relaxing procedures increasing by 60% from 2012 to 2019^25^. In our study, neurotoxin injections were the most preferred cosmetic procedures, particularly among active smokers. In addition, mesotherapy for hyperpigmentation was more common in this group. Given the widespread desire to maintain a youthful appearance and prevent aging, leveraging the information that smokers are more likely to undergo cosmetic procedures, particularly neurotoxin applications, in smoking cessation campaigns could be an effective strategy to encourage individuals, especially young people, to quit smoking.

Several dermatological features significantly predicted smoking status. Notably, skin spots in the eyebrow and shoulder areas were associated with higher smoking odds, while good skin quality in the forehead, eye area, cheeks, and neck, was linked to reduced smoking likelihood. These findings suggest that visible skin characteristics may serve as markers for smoking behavior.

### Strengths and limitations

The strengths of the study include its diverse participant pool, encompassing all age groups and both genders, and its relatively large sample size. In addition, detailed analysis of skin quality, blemishes, and wrinkles across 12 different parts of the body, along with total skin scores, added depth to the analysis. The inclusion of e-cigarettes and hookah in the analysis broadened the scope of the study. Lastly, the threshold value of skin quality deterioration determined in this study could facilitate artificial intelligence applications for evaluating skin health, enabling timely preventive measures.

This study has several limitations that should be taken into account when interpreting the findings. Firstly, the evaluation of skin quality, blemishes, and wrinkles was based on self-reported data rather than objective clinical measurements. This reliance on subjective assessment may have introduced self-reporting bias, as participants could interpret and rate similar conditions differently. Another important limitation is the cross-sectional design of the study, which restricts the ability to establish causal relationships between the variables examined. The exclusion of individuals with incomplete data may have introduced selection bias, potentially affecting the internal validity of the results. Moreover, although statistical adjustments were performed, the possibility of residual confounding cannot be ruled out, as not all relevant confounding factors may have been fully accounted for.

The study was conducted at a single center, which limits the generalizability of the findings to broader populations. Multi-center studies are needed to enhance the external validity of the results. Furthermore, some participants who used electronic cigarettes or hookah were current or former cigarette smokers, making it difficult to isolate the independent effects of these products from those of traditional tobacco use. Another limitation is the lack of detailed information on whether participants used cigarettes, electronic cigarettes, and hookah concurrently or in isolation. Lastly, the relatively low number of participants who used or quit e-cigarettes may have limited the statistical power of the analysis regarding these variables. This low participation rate is likely attributable to the legal ban on the sale of e-cigarettes in the country.

## CONCLUSIONS

This study examined the effects of smoking and other tobacco products on skin quality, skin care routines, and approaches to minimally invasive cosmetic procedures. Active smokers exhibited poorer perceived skin quality and a higher frequency of wrinkles and blemishes in all body areas evaluated. Adverse effects of e-cigarettes and hookah on the skin were also observed. Smokers were more likely to have a dry skin type and sought neurotoxin treatments and mesotherapy more frequently. We consider that highlighting the negative effects of smoking on the skin, alongside its well-known role in triggering numerous diseases, including many organ cancers, could be beneficial in smoking cessation efforts.

## Supplementary Material



## Data Availability

The data supporting this research are available from the corresponding author on reasonable request.
